# “It takes time to see the whole picture”: patients’ views on improvement in cognitive behavioral therapy and psychodynamic therapy after three years

**DOI:** 10.3389/fpsyt.2024.1342950

**Published:** 2024-03-15

**Authors:** Anders Malkomsen, Jan Ivar Røssberg, Toril Dammen, Theresa Wilberg, André Løvgren, Randi Ulberg, Julie Evensen

**Affiliations:** ^1^ Division of Mental Health and Addiction, Oslo University Hospital, Oslo, Norway; ^2^ Institute of Clinical Medicine, University of Oslo, Oslo, Norway; ^3^ Department of Psychiatry, Diakonhjemmet Hospital, Oslo, Norway; ^4^ Nydalen Outpatient Clinic, Oslo University Hospital, Oslo, Norway

**Keywords:** depression, cognitive therapy, psychodynamic therapy, qualitative research, retrospective analysis, patient perspectives, thematic analysis

## Abstract

**Introduction:**

There is a lack of qualitative research that retrospectively explores how patients with major depressive disorder view their improvement in psychotherapy.

**Methods:**

Fifteen patients who received short-term cognitive behavioral therapy and psychodynamic therapy were individually interviewed approximately three years after completing therapy.

**Results:**

Some patients had altered their views on therapy, especially those who initially were uncertain of how helpful therapy had been. They said they did not realize the extent and importance of their improvement in therapy before some time had passed, which can be explained by the surprising cumulative effects of seemingly small changes.

**Discussion:**

This should make retrospective qualitative research an important part of future psychotherapy research.

## 1 Introduction

In his novel on the mysteries of human memory, “In Search of Lost Time”, Marcel Proust wrote: “Remembrance of things past is not necessarily the remembrance of things as they were.” (1) Even though it is true that our memories fade with the passage of time, our memories also change and evolve (2). Being in therapy and improving from depression are both complex processes that may take a long time to make sense of. In addition, new experiences after therapy may stimulate new memories or interpretations of what happened during therapy. This makes it possible that some patients may view their therapy in a different light some years after its completion. Even so, very few studies have explored how patients view their improvement in therapy more than three months after treatment.

Psychotherapy is a multifaceted process in which many factors probably interact synergistically. These factors are commonly categorized into two groups: common and specific factors, with ongoing debate regarding their respective significance (3). Common factors in psychotherapy are generally regarded as the components shared among all approaches. These encompass establishing a therapeutic environment founded on trust, mutual agreement between therapist and patient on treatment rationale, a coherent understanding of psychological ailments, anticipation of improvement, and cultivation of a strong therapeutic alliance (4). Specific factors are the unique components of a particular therapeutic approach that is thought to contribute to its effectiveness. In cognitive behavioral therapy (CBT) the treatment focus is on restructuring maladaptive thoughts and behaviors through interventions focused on the here and now, like behavioral experiments (5). In psychodynamic therapy (PDT), the treatment focus is on childhood experiences, unconscious conflicts and relational dynamics, which is typically explored by interpretation and transference analysis (6).

Both CBT and PDT are theorized to alleviate symptoms of depression through common and specific factors. However, a review conducted by Cuijpers et al. concluded that neither common nor specific factors can be considered empirically validated (7). Although we don’t know exactly how psychotherapy works, we do know that it is an effective treatment for depression (8). A network meta-analysis compared the eight most common types of psychotherapy for depression in terms of efficacy, acceptability and long-term outcomes, and found CBT and PDT to be equally effective after 12 months (8). However, the authors conclude that not enough long-term studies concerning the outcome of therapy (e.g. reduction of symptoms) exist to draw any conclusions about the effects of therapy after more than one year. Unfortunately, the same is the case for qualitative psychotherapy research, where the majority of studies is based on interviews with patients conducted just a few weeks or months after therapy has ended.

Although short-term qualitative research addressing patients’ experience of psychotherapy is still rather limited, the body of research has grown in the last decades, resulting in at least three qualitative meta-analyses and a qualitative meta-synthesis (9–12). These have shown that patients across different therapy models, formats and delivery types mostly find the same elements helpful, supporting the importance of common factors. Levitt et al. identifies improvement in therapy as a process involving several elements including curiosity, engagement in pattern identification, narrative reconstruction, internalization of positive messages from the therapist, development of self-awareness and a sense of agency (10). McPherson et al. find many of the same elements as important for improvement, and highlights the importance of self-acceptance, self-knowledge and getting techniques to manage everyday thoughts and feelings (11). Ladmanová et al. also find that improvement can be brought about by many different factors related to the patient-therapist-relationship, mostly overlapping those of Levitt et al., such as feeling heard, understood, accepted, empowered, supported, safe and personally connected with the therapist (12).

To the best of our knowledge, only one study has been conducted on how patients view their improvement in short-term PDT for depression more than three months after treatment. In a study by von Below, 17 young adult patients in individual or group psychotherapy with major depressive or dysthymic disorder were interviewed at termination and after 1,5 years (13). The patients described their experience of overcoming depression as a fourfold process of feeling better, finding oneself, finding one’s way of life and viewing life differently. Also, how they viewed therapy remained generally stable over time. However, the curative aspects of other treatments (e.g. medication and yoga) were stressed by twice as many patients at follow-up, and there was a marked increase in the number who stressed the importance of existential aspects, such as finding oneself and one’s way of life.

As to CBT, only a few studies have been conducted more than three months after therapy, and these studies mainly focus on providing knowledge on how patients utilize what they learned in therapy (14–16). Glasman et al. interviewed nine patients roughly six months after they received CBT for depression to explore how they applied the helpful elements after completing therapy (14). Most patients still used a range of techniques and principles from therapy, like making self-efficacy statements or imagining what the therapist would say to them. The same was found by Berg et al. in a qualitative study of adolescents (15). French et al. interviewed 20 patients four years after they attended at least 12 sessions of CBT as treatment for depression (16). They found that patients who viewed CBT as a learning process (“Learners”) used specific skills from therapy to manage their depression after treatment ended, but patients who simply viewed therapy as an opportunity to talk about their problems (“Talkers”), did not utilize any of the skills they had been taught.

Levitt and Maxwell argue that qualitative methods serve as a valuable complement to quantitative research on mediators, thereby enhancing the exploration of causation in psychotherapy (17). While there is a growing appreciation of qualitative research, there is still a lack of retrospective research (18). Even though remembrance of things in the past is not necessarily the remembrance of things as they were, these memories are still vital to our understanding of how therapy continues to affect patients long-term. Additionally, the retrospective view may add some nuances that are lost when patients have not had the time to make full sense of their experience of being in therapy.

In the current study, we used qualitative methods to explore how patients with major depressive disorder (MDD) treated with CBT or PDT look back at their improvement process in therapy. Our research question was: How do patients view at their improvement in therapy three years after the treatment ended?

## 2 Materials and methods

### 2.1 Design, ethics and data collection

This study is part of the ongoing Norwegian project on Mechanisms of Change in Psychotherapy (MOP) (19). The aim of this randomized controlled trial is to examine moderators and mediators in CBT and PDT for 100 patients with MDD to develop a better understanding of what works for whom and how. The CBT consisted of 16 weekly sessions followed by three monthly booster sessions, and the PDT consisted of 28 weekly sessions. Treatments took place at two public psychiatric outpatient clinics in Oslo, Norway. The clinics are part of the specialist health care system and all patients are referred by a doctor.

The Central Norway Regional Ethics Health Committee (REC South East 2016/340) approved the MOP study, including the qualitative interviews. Clinical Trial gov. Identifier: NCT03022071. Informed written consent was obtained from all participants.

### 2.2 Participants

Patients were recruited from the MOP project and invited to a qualitative in-depth interview. We invited all patients who ended therapy more than three years ago and had completed follow-up after one year. A total of 20 patients fulfilled these criteria and were invited to participate via text-message. Of those who were invited, 15 answered and agreed to participate. Nine of the patients received CBT and six received PDT.

The inclusion criteria were MDD according to the DSM-IV (based on a clinical interview and MINI), age 18–65 years, the ability to understand, write and speak a Scandinavian language, and the ability to give informed consent (20). Exclusion criteria were current or past neurological illness, traumatic brain injury, current alcohol and/or substance dependency disorders, psychotic disorders, bipolar disorder type 1, developmental disorders and intellectual disability.

There were 11 female and four male patients, all between 20 – 50 years old, with a median age of 34. None of the patients had previously been admitted to a psychiatric hospital, but eight had previously received psychotherapy. Eleven patients had previously experienced at least one episode of depression. Two patients were diagnosed with at least one personality disorder (PD) at baseline, one with avoidant and dependent PDs, and one with an avoidant PD, according to the Structured Clinical Interview for DSM-IV Axis II (21). Four patients were diagnosed with panic disorder with agoraphobia, one with social anxiety, one with generalized anxiety disorder and one with dysthymia. One patient dropped out of therapy due to dissatisfaction.

At baseline, the mean level of depression was 17, measured by Hamilton depression rating scale (HDRS) (22). Seven patients used antidepressants. After 28 weeks, the mean level was 12. Ten patients had improved their score compared to baseline, two patients remained stable (at 17 and 19) and three patients had increased their score (more depressed). After one year, the mean level of depression was 7. Thirteen patients had now improved their score compared to baseline, one patient remained at an unchanged score and one patient had an increased score. The patient with a stable score was diagnosed at baseline with recurrent major depressive disorder, generalized anxiety disorder and panic disorder with agoraphobia. The patient with increased score was diagnosed at baseline with major depressive disorder without comorbidity.

After three years, the mean level of depression was 10. Seven patients reported that they had received psychotherapy outside the MOP study during the last three years. Four patients still used antidepressants. Compared to the HDRS score after one year, five patients continued to improve their score, six patients had an increased score (more depressed) and one patient remained at a stable score. Of the six patients who got more depressed from one to three years, all were diagnosed with either recurring depressive disorder or dysthymia at baseline, one had avoidant personality disorder and four had a co-morbid anxiety disorder. There were no significant differences in depression severity between the CBT and PDT at any time point.

### 2.3 The interview

All patients were interviewed during the course of a few weeks. The interviews were performed by the first author (twelve interviews) and the fifth author (three interviews). There were no personal connections between the patients and interviewers, and neither of the interviewers have acted as therapists in this study. To make sure that the patients felt free to share their criticism and be honest if therapy had not helped them, they were informed that the interviewers were blinded to the identity of their therapist. Before the interviews were conducted, the participants were explicitly told that we were interested in both good and bad experiences of therapy. A research assistant transcribed the interviews and anonymized all the transcriptions. In a previous study embedded in the MOP project, seven of the patients were interviewed about their experience of being in therapy just a few weeks after their therapy ended (23).

A semi-structured interview was designed specifically to explore our research question. Several follow-up questions were asked to ascertain whether patients attributed improvements to therapy or other influences. In addition to the helpful elements, we also wanted to explore the unhelpful and harmful elements of therapy to get the full picture of the patients’ experiences. First, the patients were questioned about how they currently were feeling and what (if any) psychiatric treatment they had received after finishing therapy in the MOP study. Second, they were asked how they experienced the therapy they received three years ago, if they remembered any particularly important episodes from the treatment, what they found helpful, how (if) they experienced improvement, and what they found difficult or less helpful in therapy. Third, they were asked about the importance of metaphors in their therapy, and the importance of some of the metaphors we identified in our previous studies on therapeutic metaphors (tools, depth and depression as a monster) (23, 24). The questions on metaphors were included because we previously identified them as important for patients’ experience of improvement in therapy. Examples of questions are found in **Table 1**.

**Table 1:** Examples of questions from the interview with patients.

General questions from the interview	Specific questions from the interview
How have you been during the three years after your therapy ended?	Have you sought out any other psychiatric treatment? Why not? If so, how did you experience the treatment? Why did you choose that kind of treatment?
How did you experience the therapy you got three years ago?	What did you find helpful in therapy? How did it help you? What did you find less helpful? Did you find any aspects of therapy harmful? Have your views on the therapy changed during these three years? Do you remember any important situations, events or episodes from therapy that was especially meaningful or important to you?
Did you change in any way during the treatment?	Did your thoughts change? Your feelings? Did you do things differently? Did your relationships change? Did you understand something new about yourself?
What did metaphors mean to you during your therapy?	Do you remember any verbal images or metaphors from therapy that became important to you? Was it important for you to get tools in therapy? Was it important for you that your therapy was deep? What does it mean to you that a therapy is “deep”? Some describe depression as having monster inside, did you feel that way?

### 2.4 Therapists and treatment

The therapists in MOP were psychiatrists, psychologists and psychiatric nurses. All had a minimum of two years of training in CBT or PDT. In addition, they had a one year training period on the principles of CBT or short-term PDT before receiving patients in the study. Experienced supervisors monitored their adherence to the treatment principles in weekly or biweekly group supervisions throughout the therapy period. All therapy sessions were recorded, and the recordings were reviewed with focus on the initial phase of treatment, case formulation, individual treatment strategies and termination of therapy.

Patients received therapy according to the principles of either CBT or PDT. The principles of therapy in the CBT group were based on the book “Cognitive Therapy of Depression” by Aaron Beck et al. (5). All CBT therapists made a case formulation based on cognitive principles together with their patients. The therapists helped patients recognize negative, automatic thoughts; examine evidence for and against these thoughts; substitute unhelpful thoughts; recognize the connections between cognition, affect and behavior; in addition to identify and alter dysfunctional beliefs. The therapy focused on the “here-and-now” and limited attention was paid on recollecting the past (5). The treatment manual advised therapists to be continuously active and facilitate collaboration, and to create a working alliance with the patient.

The principles of therapy in the PDT group were built on the general psychodynamic principles described in the book “Long-term psychodynamic psychotherapy” by Glen O. Gabbard (6). The time-limited design was built on the principles described by Høglend et al. (25). All therapists were asked to make a case formulation together with their patients based on PDT principles. Therapists were also encouraged to explore sensitive topics, explore the patient-therapist relationship, address transactions in the relationship, use material about interpersonal relationships outside therapy as the basis for interventions, encourage exploration of thoughts and feelings about the therapy, and interpret direct manifestations of transference with moderate intensity (26).

### 2.5 Analysis

Thematic content analysis was used to analyze the material (27). We identified themes or patterns within the data by using an inductive bottom up-approach (28). Before we conducted the interviews, we speculated based on previous research that there would not be any substantial differences in how CBT and PDT patients viewed their therapy.

The first and last author read all the transcripts looking for answers to the research question. We also coded what patients experienced as unhelpful and harmful in therapy, but excluded these codes in the final thematic analysis. This was not done to exclude criticism per se, but to be able to go in-depth on the views on improvement, which is the main aim of this study. However, we have included critical comments from patients when they are regarded as relevant to the elements that were found helpful – e.g. if an element found helpful by some is not found helpful by others, or when patients say that they did not receive enough of something they found helpful.

The first and last author familiarized themselves with the data, generated codes and searched for themes. The second author gave feedback on the first and last author’s coding and themes. The first author refined the initial coding and themes before sending the most relevant interview parts, the coding and the suggested themes to all authors. All authors discussed their unique understanding of the material, and criticized the coding and themes. The first author then adjusted the themes after getting feedback from the other authors. Finally, all authors agreed on the current thematic categorization and naming of themes. This process made our interpretations less dependent on individual biases and blindspots (29). We wanted the analysis to be based on the patients’ experience of improvement, and were thus blinded for any quantitative data regarding the outcome of the patients during interviews and analysis. After the analysis, we looked at the quantitative data to better inform our discussions of the results. However, did this not change our themes or subthemes.

We indicate the recurrence and representativeness of patients’ experiences by using the labels general, typical and variant as suggested by Hill et al. (30). When something is mentioned by all patients (or all but one) it is labeled as general, in the text referred to as “all patients”. Something is considered typical when it is mentioned by half or more than half of the patients, referred to as “most patients”. We use the expression “some patients” when something is found to be a variant represented by less than half the patients, either in both groups or in one of the groups (if specified). The abbreviations CBT and PDT will be used to specify the therapeutic approach. When no abbreviation is used, it means that patients from both groups are included.

The authors have different therapeutic orientations. J.E., T.D. and J.I.R. are CBT therapists, T.W. and R.U. are PDT therapists. A.L. and A.M. have no specific therapeutic orientation. We make this transparent in accordance with the checklist of reporting qualitative research by Tong et al. (31)

## 3 Results

We identified three main themes: “Helpful elements in therapy”, “Experienced improvement” and “The significance of the passage of time”. In total, 15 subthemes were identified. These are summarized in **Table 2**.

**Table 2:** The identified themes and subthemes.

Themes	Subthemes	Examples of quotes
Helpful elements in therapy	Being taken seriously	“I realize now that this isn’t just something I imagine.”
	Expressing thoughts and feelings	“As soon as I said it out loud and it left my mouth, I got another impression of those thoughts and feelings.”
	From chaos to order	“I felt that the therapy cleaned up and sorted in my chaos of feelings.”
	Getting therapeutic tools	“She said you can set aside 20 minutes each day for negative thoughts. That became a tool that I have brought with me.”
	Digging deep	“You spend a long time on something, you dig and you twist and you turn.”
	Reinterpreting childhood	“I suddenly realized my childhood had not been as perfect as I thought.”
Experienced improvement	Improving self-worth	“I believed more in myself, and that I wasn’t as weird as I thought.”
	Accepting oneself	“I think the treatment made me accept that maybe I have to take things a bit slower.”
	Opening up	“It became more and more important for me to be able to show all my emotions, not just the good ones.”
	Setting boundaries	“I’m much better at being less of a puppet.”
	Taking responsibility	“It made me feel that I have a responsibility to take hold of myself.”
The significance of the passage of time	Much forgotten, but still important	“Even though I don’t remember anything, that doesn´t mean it wasn´t important to me.”
	Towards a more positive assessment of therapy	“It has helped me more than I thought after I finished.”
	Therapy as a steppingstone	“I think the biggest change has happened after I finished therapy.”
	Gaining distance from depression	“In retrospect, it feels like I was a different person than myself at that time.”

### 3.1 Helpful elements of therapy

#### 3.1.1 Being taken seriously

Most patients in the PDT group and some in the CBT group said that the experience of being taken seriously helped them improve. However, patients differed in what made them feel that they were taken seriously. One patient (PDT) remembered how her therapist said that she felt a bit sad when looking at her, and that this made her realize that her sadness was “serious”, which in turn made her take herself more seriously. Before this happened, the patient said she “didn’t treat it as serious as it actually was”. When she experienced that her therapist took her suffering seriously and was affected by it, the patient also changed her own views. This realization motivated her to engage in therapy.

Another patient (PDT) said that being taken seriously in therapy made her feel safe, and that being in therapy also made others take her problems more seriously: “I could tell my mum and dad that I’m actually in therapy with a psychologist now. It’s not … it’s not just me making stuff up here. It’s not just that I don’t want to get up in the morning.”

One patient (CBT) said that many of her friends became afraid when she told them about her suicidal thoughts. The fact that her therapist did not get afraid, made her feel that she was taken seriously: “Not to be met with fear, but to be met *for real*, with ‘these are serious thoughts, and I take it seriously’. To feel that you are met on an interpersonal level that is not about you scaring someone. That reassured me a bit.” Another patient (CBT) said that he experienced improvement even before therapy started. He felt that he was being taken seriously because he was referred to the outpatient clinic: “I think that just knowing you’re going to get treatment by itself may give some improvement … That feeling of getting help. That made me feel like I was being taken seriously.”

#### 3.1.2 Expressing thoughts and feelings

Most patients said that just being able to express their thoughts and feelings was helpful in itself. One patient (CBT) said that she got a new impression of her thoughts and feelings as soon as she said them out loud: “It became more distant, like `No, I don’t really think that way. I don’t really want to die.´” Another patient (PDT) said that having the opportunity to say things (“to air it out to an actual person”) was important for her: “It’s liberating to be able to be that honest. You can’t really tell everything that goes on in your head to family and friends, at least I can’t.”

#### 3.1.3 From chaos to order

Some patients said that therapy helped them by either “sorting” or “cleaning” their thoughts and feelings. This theme may be a result of being able to express thoughts and feelings, but the active role of the therapist seemed more important in this process of organizing. All patients mentioned that the therapist helped them sort or clean – most often by asking clarifying questions. One patient (CBT) said that the therapist assisted her in creating some order in a chaos of emotions, which gave her a sense of control: “It went from chaos to a bit more control, as I became more aware of which feelings I actually had.” Another patient (PDT) said that therapy helped her to “clean up” her memories of her childhood: “I don’t think I would be satisfied if I didn’t go all the way back, to clean up all the mess there.”

#### 3.1.4 Getting therapeutic tools

Most patients in the CBT-group and some patients in the PDT-group said that it was important for their improvement that they got “tools” in therapy. However, the patients defined tools in many different ways and gave very different examples, such as to “place responsibility where it belongs” (CBT), “deal with emotions” (CBT), “set boundaries” (CBT), “tips to fall asleep faster” (CBT), “think more about my positive sides” (CBT), “ask myself the why-question” (CBT and PDT), “how to speak to my parents” (PDT) and “question the origin of my thought patterns” (PDT).

One patient (CBT) defined a therapeutic tool in the following way: “A tool is a strategy for how to handle feelings, thoughts or psychical reactions. Tools are very, very specific, and are impossible to misunderstand.” This patient was satisfied with the therapy overall, but still felt that therapy did not provide her enough tools. She was sure that she would have improved more if she got more tools, and wondered if her therapist perhaps denied her such tools on purpose. She also expressed some ambivalence towards tools: “In hindsight, I have wondered … if you just get some tools, are you still able to work on a deeper level, or do you just put a lid on stuff?”

#### 3.1.5 Digging deep

Most patients in the PDT-group and some patients in the CBT-group said it was important for them that the therapy was “deep”. Most patients seemed to think that going deep meant one of two things: to explore childhood memories (most common in PDT-group), or to explore patterns of thoughts and feelings (most common in CBT-group). One patient (CBT) said it was not important for him to talk about his childhood, but rather to look closely at his thoughts and feelings here-and-now: “I think we went deep by trying to understand my way of thinking and feeling. We didn’t just scratch the surface.” Another patient (PDT) said it was important for her to understand why she thought and felt the way she did, and that the answers could be found by digging back in time: “It was important for me to understand why I have these patterns of reacting … I think you get that by talking about childhood and early experiences.”

#### 3.1.6 Reinterpreting childhood

Some patients in both groups of therapy said they altered their views on their own childhood after talking about it in therapy. Typically, the patients came to view their childhood as more problematic than they originally thought. One patient (CBT) said the therapist challenged her view that her upbringing had been normal: “She turned my experience of what had happened around. When I told her an episode from my childhood where I thought something was my fault, and that I was the problem, she put the responsibility … or reminded me, in a way, that the responsibility should be put elsewhere.” Another patient (PDT) was surprised by the importance of her childhood experiences: “It’s funny how the mind and memory works … How you judge something based on what you have experienced just once.” This realization of the importance of childhood experiences was described as “liberating” for the patient, and made it easier for her to question and change some of her beliefs.

### 3.2 Experienced improvement

#### 3.2.1 Improving self-worth

Some patients said that their self-worth improved in therapy. One patient (CBT) said her self-worth improved as a result of placing less responsibility upon herself for her troubled childhood. This improvement also made it easier for her to believe the compliments she got, which further improved her belief in herself. Another patient (PDT) said that the therapist’s curiosity and lack of condemnation made her feel more confident: “I believed more in myself, and that I wasn’t as weird as I thought.” For her, believing in yourself and having trust in your own value as a person is “what it’s all about” in therapy.

#### 3.2.2 Accepting oneself

Some patients said that therapy made it easier for them to accept themselves. One patient (PDT) said: “I think the treatment made me accept that maybe I have to take things a bit slower.” Another patient (PDT) learned to accept her sexuality without shame because she felt accepted by her therapist: “I didn’t feel condemned. I felt that she was open, and there was almost like a joyfulness in the room.” One patient (CBT) who had a somatic disease that prevented him from working, said therapy made him “come to terms with the situation”, making him less frustrated and bitter.

#### 3.2.3 Opening up

Some patients said that therapy made it easier to be more open about their struggles. One patient (CBT) said that talking to the therapist also made it easier to be open with friends and family: “It became more and more important for me to be able to show all emotions, not just the good ones … To dare to say that things are difficult, that changed for me. It lessened my fear of how it would be received.” Another patient (PDT) also found it easier to show her emotions to others, and this openness improved her relationships: “Because I’m better able to show that I care about someone, all my friendships are strengthened.”

#### 3.2.4 Setting boundaries

Some patients said that therapy helped them set more boundaries for themselves and others. One patient (CBT) said that her new boundaries came with some relational consequences: “I think it contributed to more frustration for my mother and sister, because I started to say ‘no’. In general, it has been quite wonderful. Really. To not always do something when you don’t really want to.” Another patient (CBT) said that therapy had made it possible for him to distance himself from his parents’ control: “I’m much better at being less of a puppet.” The therapist’s support in separating from her parents was also important for a patient (PDT) who moved away from home as a result of therapy: “When I moved out, I could finally start working on myself, and put everyone else aside for a moment.”

#### 3.2.5 Taking responsibility

Most patients in CBT and some in PDT said that being in therapy made them realize that they are responsible for their own life. One patient (CBT) said that therapy convinced him that he was free to do what he wanted, but also responsible: “What I realized after talking to her (the therapist) was that, at the end of the day, it’s up to me what I think and mean about things. It’s nobody else’s responsibility than mine that I’m feeling fine.” Reflections upon responsibility was less frequent in the PDT-group, but one patient said she now appreciated the importance of taking responsibility. She believed this realization, which came as a result of her buying an apartment and getting a job, made her benefit more from a new course of therapy she got after the MOP-project ended: “I think the biggest difference in the new therapy was that I realized that I have do the work myself. Maybe that’s the biggest difference, really.”

### 3.3 The importance of time passing

#### 3.3.1 Much forgotten, but still important

Many patients said they struggled to remember specific details about the therapy, especially at the beginning of the interview. To explain their lack of remembrance, many patients said they had not thought much about therapy after ending it. One patient (CBT) said he had “erased” the whole period of depression from his memory: “It was really bad, it lasted a long time, and I don’t think about it. What I´m thinking about now? How I want my future to be.”

Few could remember exactly what was said during their therapy, but instead remembered the experience of being in therapy – and certain important moments. However, the lack of detailed memories did not necessarily mean that the therapy was not important to the patients. One patient (PDT) said: “Even though I don’t remember anything, that doesn´t mean it wasn´t important to me.”

#### 3.3.2 Towards a more positive assessment

Most patients had not changed their views on therapy in radical ways, but some said they had gotten new perspectives on both their depression, therapy and improvement. As one patient (CBT) put it: “It takes time to see the whole picture”. In those cases in which there was a change of view, it was towards a more positive assessment.

Some patients in the CBT-group said that they did not realize until later how important the changes they experienced during therapy would become. One patient (CBT) said that “right after therapy I don’t think I realized what I had actually learned”, but “now, when I feel that I’m heading into a depression, I’m able to use those techniques”. He also remembered a moment when his therapist challenged him by questioning his way of thinking. His parents advised him not to get another girlfriend, and he felt obliged to listen to them: “She (the therapist) was like: ´Why?´ I remember that very clearly: ´Why can´t you just think for yourself?´” He said this way of thinking had later helped him to start working again and make other important changes in his life.

Another patient (PDT) initially felt that the therapy had ended too early: “I knew that I got great help by coming here. At the same time, I was like ‘do they let me go too early, since I always crash again after a few months?” Now, after some years had passed, she concluded that therapy had not ended prematurely. This view was also shared by another patient (CBT) who was afraid she would become dependent on the therapist if she continued any longer. She said: “In hindsight, I see that if I had continued and continued and continued in therapy, I wouldn’t be able to say ‘I will manage it myself; I am the one who is going to face these thoughts.”

#### 3.3.3 Therapy as a stepping stone

Some patients said they changed more after therapy ended than during the actual therapy. However, the subsequent change was still experienced as a consequence of therapy. One patient (PDT) said: “Previously, I was really insecure and shy, with a lot of anxiety, and pretty depressed at times. Gradually, something just loosened.” She attributed this change to changes in her life during therapy (moving to a different place), after therapy (breaking up with a “bad” boyfriend) and the therapy itself: “Yeah, I think that was the perfect combination.” Even though it happened afterwards, she believed that the courage to break up with her boyfriend came from therapy.

Another patient (CBT) also said that the biggest changes happened after she ended therapy. She got some “tools” in therapy that she used to change her life, and therapy played the role as a starting signal for this change. The patient used a metaphor of cycling to describe this: “I feel that therapy provided some supporting wheels, but I have to learn to ride myself. That’s when the biggest change happens.”

#### 3.3.4 Gaining distance from depression

Most patients had distanced themselves more from their depressed state. One patient (CBT) said that while she initially felt that she was herself when she was depressed, she now thought differently: “Afterwards, I feel like it was another person, an inner voice or a monster, but at the time I felt like ´This is me´”. She viewed this change in perspective on her depression as a sign of improvement.

Another patient (PDT) said she believed depression was part of her identity while in therapy. Her brother later introduced her to the idea that depression may also be something you could have: “I think that could have helped me at the time, that I *had* a depression … I now think that’s a better way of looking at it.” One patient (CBT) said that the process of accepting the severity of her depression had been a long one. It helped her when another therapist (two years later) told her that a depression can be a kind of trauma: “That helped me acknowledge that I was actually … in a bad place. Looking back, I don’t recognize myself. But that’s okay. It’s not something I need to push away or be ashamed of.”

## 4 Discussion

This study aimed to explore how patients look back at their improvement in therapy three years after completion. Patients viewed their improvement from depression as a result of increasing their self-worth, accepting themselves, opening up to others, setting boundaries and taking responsibility for their own mental health. This was facilitated in therapy by being taken seriously, expressing difficult thoughts and feelings, creating order within inner chaos, getting therapeutic tools, digging deep into past experiences or patterns of thoughts or feelings, and reconsidering childhood experiences as being more problematic than initially perceived. Even though they said their views on therapy had been mostly stable during the three years, some had moved towards a more positive assessment of therapy, and some said that time’s passing had made them realize how important their therapy had really been. We will now discuss some of these results in light of previous research.

### 4.1 Significance of the passage of time

Most patients had not changed their views on therapy in fundamental ways, but the passage of time had brought about a change in perspective for some patients. This in line with that was found by von Below et al. in their follow-up of young adults (13). However, we have to be cautious in any further comparisons between the patients in our and their study, as the mean age in their population was 22 years (young adults) and ours was 35 (adults). This age difference may explain why we don’t see the same importance of “becoming older” and “finding one’s way of life” in our patients.

Most patients who said they had changed their views on the depression, had gained more distance from their depressed state. As we know that an episode of major depression usually lasts several months regardless of how it is treated, it is no wonder that it may take years for patients to distance themselves from it (32). It should be noted that most patients experienced this increased distance as a sign of improvement. This is important because we previously identified that some therapists (especially in PDT) are reluctant to support this way of distancing oneself from depression, such as viewing depression as an opponent or something foreign (e.g. as a disease) (24). In light of this, it is interesting that one patient who received PDT said this way of conceptualizing depression was not introduced during her therapy. When it was later introduced to her by her brother, she found it helpful. This does not imply that one way of conceptualizing the self-depression-relation is better than another. Rather, it could be seen as an inspiration for therapists to be more interested in exploring different conceptualizations of depression with their patients.

Some of the patients said they had moved towards a more positive assessment of their therapy during the three years, especially those who initially were uncertain of how helpful therapy had been. The Hamilton depression scores support their experience, as several patients continue to improve after therapy has ended. This could inspire therapists to tell their patients that it may be common to underestimate the effects that small changes in therapy may have in the long run – exemplified by the patient who viewed therapy as important, but still a mere “stepping stone” for her long-term improvement process. Another important finding is that several patients had changed their views on what they previously regarded as a premature termination of therapy. One patient even raised concerns as to whether she could had gotten dependent on therapy had it not been terminated as planned, and was now thankful that it ended when it did. By realizing that the end of therapy may only be the beginning of the improvement process, it may also be easier for therapists to end therapy before the patient is in complete remission – something that is often necessary in a health care system with limited resources.

### 4.2 Identifying synergistic and cumulative effects

We have previously shown that the improvement process is experienced after therapy as a gradual, non-linear and slow process involving many elements that interact synergistically (33). Patients seldom refer to just one particular element of therapy – rather, “it’s the whole package” that stimulates improvement. After three years, the therapeutic improvement is still experienced as synergistic, with even more elements that together constitute “the package”.

The process of change was not experienced as sudden, but as a gradual process in which each element strengthened the others. This positive reinforcement of therapeutic elements is what we refer to as therapeutic synergy. All patients with a successful outcome (continued improvement) mention several contributing factors that seemingly work synergistically. An illustrative example of this synergy is found in the improvement of a patient (PDT and steady psychometric improvement) who said she experienced more change after therapy ended than during the actual therapy. First, intra-therapeutic elements made her less anxious and made her depressive symptoms less of a barrier to change. Second, extra-therapeutic elements like moving to another place was also experienced as important for improvement, but not related to the therapy. Third, another important element was that she broke up with her boyfriend sometime after therapy. This could be thought of as a post-therapeutic element as the patient clearly states that she experienced it as a consequence of therapy (e.g. because her increased belief in herself gave her the necessary courage to do it). It may be that patients with a less successful outcome fail to generate the synergy that seems so vital for the success of those who have a stable reduction of their depressive symptoms, but more research is needed to clarify this.

Post-therapeutic elements are probably understudied as a result of the rarity of retrospective qualitative studies. This may undervalue some of the contributions of therapy, especially those elements that make it possible for patients to make definitive changes in their life after therapy. Examples of such elements may be those that our patients remember as most important for their improvement when they look back at therapy: improving self-worth, accepting oneself, using therapeutic tools, opening up to others, setting boundaries and taking responsibility. If therapists focus on those elements in therapy, the chances of generating a synergy with post-therapeutic elements may increase. However, these elements are also mentioned as important by patients who relapse or do not remit – thus, they seem to be necessary, but not sufficient by themselves.

The retrospective view on therapy also allows us to appreciate the importance of cumulative therapeutic effects. One can say that a small, but repeated change may have a great cumulative effect that exceeds initial expectations. As an illustrative example of cumulative effects, one patient (CBT and stable psychometric improvement) said that he was unsure whether what he learned in therapy had been useful. For him, the habitual act of questioning his own thoughts was later what he experienced as most important from therapy. He said that this way of questioning his own thoughts still helped him to avoid falling back into depression, and that it also helped him to get back to working after therapy. This may inspire therapists to focus more on changes that can have cumulative effects and continue to facilitate improvement after therapy. Identifying these elements is an important task for psychotherapy researchers and is a strong argument for more retrospective and long-term research.

Not all patients continued to improve their score after one year. Compared to the HDRS score after one year, five patients continued to improve, six patients became more depressed and one patient remained at a stable score. When we compare these scores with the qualitative interviews, it is clear that all patients who continue to improve experience some kind of synergy between intra-, extra- and post-therapeutic factors. The most obvious difference between those who experience synergy and those who do not, is co-morbidity. Of the six patients who got more depressed from one to three years, all were diagnosed with either recurring depressive disorder or dysthymia at baseline, one had avoidant personality disorder and four had a co-morbid anxiety disorder. Unfortunately, we have not been able to identify any other characteristics that may explain why some experience synergy while others do not. More research is needed to shed light on this important topic.

### 4.3 Diminishing importance of the therapeutic approach

The study of mediators in psychotherapy is an important way to improve our understanding of how therapy works (34). There is still ongoing debate about the importance of common and specific factors in psychotherapy, but some research support that there are specific factors mediating change in both CBT and PDT (35–37). Further research is still necessary to shed light on these issues (3, 7). To explore this, we need both RCTs, expert interviews, process-based research and qualitative studies (17, 38, 39).

This study provides new knowledge from the perspective of qualitative research. As a result of our strict fidelity control, we can be sure that patients in the CBT and PDT groups received very different therapies. Despite this, our conclusion is that there are striking similarities between the groups regarding which elements they experience as important for improvement. Metaphorically, CBT and PDT seem like two different pathways that ultimately lead to the same high road of improvement. This finding somewhat contradicts a qualitative study by Nilsson et al. which found that patients in CBT and PDT continue to improve 6-12 months after therapy, but in “clearly different ways” (40). Our results are more in line with a study by Göstas et al. where they interviewed 14 patients with unspecified diagnoses who received either CBT or PDT (41). They describe the similarities between the two groups as “striking”, and find that the similarities far outweigh the differences. The same similarities across different approaches is found in several recent qualitative meta-analyses (10–12). Our study strengthens this conclusion by showing that the same applies after three years.

In fact, the differences in how improvement in CBT and PDT is experienced seem to have diminished in the years that have passed after therapy ended. We have previously interviewed patients who received CBT and PDT in the MOP project just a few weeks after therapy ended (33, 42). After three years, patients who received CBT highlight fewer CBT specific elements than a few weeks after therapy. The patients in CBT now seem to view their improvement more broadly than they did immediately after therapy, with emphasis on the same processes that are mentioned by patients who received PDT. Learning to take responsibility is the only point in the CBT group that is clearly stated both immediately after therapy and three years later, maybe showcasing the importance of this element in CBT.

It is difficult to say whether the diminishing importance of specific elements is a result of a different interviewer, different patients, time’s passing or other circumstances. We speculate that it is due to a process in which the specific techniques, the therapist as a person and the therapeutic insights have been “integrated” into the patient, and thus been given a new meaning – no longer as a “result” of therapy, but as a “part” of the patient. After this integration has happened, the differences experienced by patients between CBT and PDT will be less clear than immediately after ending therapy.

Nilsson et al. found that the positive outcome of therapy was easier to grasp for patients in CBT than PDT (40). In contrast, our retrospective interviews revealed that patients in CBT also found that it took time before they realized the real importance of the changes they experienced during therapy. A mixed-method study by De Smet et al. also found that the positive results of therapy were clearer to patients who received CBT than PDT, but speculate that this might be explained by the fact that more patients in CBT had recovered from depression (43). In our study, there were no significant differences in recovery rates between the groups, which could be taken to support the speculation that there is a correlation between clarity and improvement.

### 4.4 Strengths and limitations

This study has several limitations. The paper explores how patients view their improvement and which elements of therapy they found helpful, but it does not explore what was actually said and done in the therapy room. It is important to remember that what patients experience and remember is not necessarily the same as what happened. Video analysis of therapy sessions could be conducted in the future in order to clarify this. Examining therapy sessions could also illuminate the significance of particular therapist skills in generating therapeutic change with cumulative effects.

Some may also argue that our analysis is limited by a narrow focus on improvement. Even though all patients said they had improved in some way or another, not all patients improved according to psychometric measures, and several patients relapsed after three years. To get a more nuanced view on improvement, we included patients that were both improved, not improved and deteriorated according to their HDRS score. Still, all the patients we interviewed look back positively on their treatment in one way or another, while there were also several aspects they found less helpful, not helpful and even some aspects they found harmful. When we focus solely on the process of improvement, a major benefit is that we are able to go into more details on this process. The cost is that knowledge about the important process of deterioration, as well as the experience of not being helped by therapy, is lost.

The number of patients in this study is also quite small. The question of adequate sample size in qualitative research is somewhat contested, and the acceptable number of included participants will always be affected by several aspects (44). The concept of saturation is widely accepted as a guiding principle in qualitative research, although still much-debated and conceptualized in numerous ways (45). We still decided to use the concept of saturation to determine how many patients should be included. The last two interviews provided no new answers, and we thus decided to stop interviewing patients after we reached a total of 15. However, one cannot be certain to have an adequate number of patients before the data is analyzed, as the answer also relies on the quality of the data and the amount of useful information obtained from each participant (46). Our analysis did not reveal an urgent need for more participants, as no new themes or codes emerged after the first twelve interviews were analyzed. A systematic review by Hennink and Kaiser found that saturation can be achieved in a narrow range of interviews (9–17) in studies with relatively homogenous study populations and narrowly defined objectives (47). Thus, we concluded that the current sample size was sufficient to answer our research question.

## 5 Conclusions

After three years, some patients had shifted towards a more favorable evaluation of their therapy, particularly those who initially doubted its efficacy. Patients perceived therapy as a mere stepping-stone to their improvement, highlighting the great cumulative impact of seemingly small changes. Therapists should consider how they can facilitate the changes that were experienced as important for long-term improvement, such as improving self-worth, accepting oneself, opening up, setting boundaries and taking responsibility. Even though patients’ views on therapy are mostly stable, the passage of time adds some important nuances. For instance, the differences between the experience of improvement in cognitive and psychodynamic therapy seem less important in retrospect. This highlights the need for more retrospective qualitative research.

## Data availability statement

The original contributions presented in the study are included in the article/supplementary material. Further inquiries can be directed to the corresponding author.

## Ethics statement

The Central Norway Regional Ethics Health Committee (REC South East 2016/340) approved the MOP study, including the qualitative interviews. The studies were conducted in accordance with the local legislation and institutional requirements. Informed written consent was obtained from all participants.

## Author contributions

AM: Writing – original draft. JR: Writing – review & editing. TD: Writing – review & editing. TW: Writing – review & editing. AL: Writing – review & editing. RU: Writing – review & editing. JE: Writing – review & editing.
